# Neurotensin and Xenin Show Positive Correlations With Perceived Stress, Anxiety, Depressiveness and Eating Disorder Symptoms in Female Obese Patients

**DOI:** 10.3389/fnbeh.2021.629729

**Published:** 2021-02-16

**Authors:** Ellen Wölk, Andreas Stengel, Selina Johanna Schaper, Matthias Rose, Tobias Hofmann

**Affiliations:** ^1^Department for Psychosomatic Medicine, Charité Center for Internal Medicine and Dermatology, Corporate Member of Freie Universität Berlin, Berlin Institute of Health, Charité – Universitätsmedizin Berlin, Humboldt-Universität zu Berlin, Berlin, Germany; ^2^Department of Psychosomatic Medicine and Psychotherapy, University Hospital Tübingen, Tübingen, Germany

**Keywords:** gut-brain axis, obesity, psychiatric, psychosomatic, sex difference

## Abstract

**Objective:**

Neurotensin and xenin are two closely related anorexigenic neuropeptides synthesized in the small intestine that exert diverse peripheral and central functions. Both act via the neurotensin-1-receptor. In animal models of obesity reduced central concentrations of these peptides have been found. Dysregulations of the acute and chronic stress response are associated with development and maintenance of obesity. Until now, associations of both peptides with stress, anxiety, depressiveness, and eating disorder symptoms have not been investigated. The aim of the present study was to examine associations of neurotensin and xenin with these psychological characteristics under conditions of obesity.

**Materials and Methods:**

From 2010 to 2016 we consecutively enrolled 160 inpatients (63 men and 97 women), admitted due to obesity and its mental and somatic comorbidities. Blood withdrawal und psychometric tests (PSQ-20, GAD-7, PHQ-9, and EDI-2) occurred within one week after admission. We measured levels of neurotensin and xenin in plasma by ELISA.

**Results:**

Mean body mass index was 47.2 ± 9.5 kg/m^2^. Concentrations of neurotensin and xenin positively correlated with each other (women: *r* = 0.788, *p* < 0.001; men: *r* = 0.731, *p* < 0.001) and did not significantly differ between sexes (*p* > 0.05). Women generally displayed higher psychometric values than men (PSQ-20: 58.2 ± 21.7 vs. 47.0 ± 20.8, *p* = 0.002; GAD-7: 9.7 ± 5.8 vs. 7.1 ± 5.3, *p* = 0.004; PHQ-9: 11.6 ± 6.6 vs. 8.8 ± 5.9, *p* = 0.008; EDI-2: 50.5 ± 12.8 vs. 39.7 ± 11.9, *p* < 0.001). Only women showed positive correlations of both neuropeptides with stress (neurotensin: *r* = 0.231, *p* = 0.023; xenin: *r* = 0.254, *p* = 0.013), anxiety (neurotensin: *r* = 0.265, *p* = 0.009; xenin: *r* = 0.257, *p* = 0.012), depressiveness (neurotensin: *r* = 0.281, *p* = 0.006; xenin: *r* = 0.241, *p* = 0.019) and eating disorder symptoms (neurotensin: *r* = 0.276, *p* = 0.007; xenin: *r* = 0.26, *p* = 0.011), whereas, men did not (*p* > 0.05).

**Conclusion:**

Neurotensin and xenin plasma levels of female obese patients are positively correlated with perceived stress, anxiety, depressiveness, and eating disorder symptoms. These associations could be influenced by higher prevalence of mental disorders in women and by sex hormones. In men, no correlations were observed, which points toward a sex-dependent regulation.

## Highlights

- Neurotensin and xenin are long-known neuropeptides with anorexigenic effects.
- Neurotensin and xenin seem to be associated with psychopathology under conditions of obesity.
- Sex-specific regulation of neurotensin and xenin was found for perceived stress and anxiety.
- Only in obese women neurotensin and xenin showed positive correlations with depressiveness and eating disorder symptoms.
- Alterations of circulating levels of neurotensin and xenin may be involved in the emergence and maintenance of obesity.

## Introduction

With a worldwide prevalence of 13.1%, which has increased threefold since 1975, obesity (body mass index, BMI ≥ 30 kg/m^2^) is a widespread and serious disease, mostly because of its physical and mental comorbidities (World Health Organization, 2017, 2020). In Europe, 23.3% of the adult population is obese (World Health Organization, 2017). The prevalence of 38.2% in the United States of America is critically high among adults (Organisation for Economic Co-operation and Development, 2017). Comorbidities of obesity include dyslipidemia and insulin resistance together with manifest diabetes mellitus type 2 and arterial hypertension (Grundy, 2002; Garg et al., 2014). In addition, mental comorbidities are associated with obesity (Petry et al., 2008; Preiss et al., 2013; Herpertz, 2015). Depressive, anxiety, somatoform, and binge eating disorders (BED) are the most frequent ones (Petry et al., 2008; Preiss et al., 2013; Herpertz, 2015). Moreover, psychosocial risk factors are involved in the etiology of obesity and obesity itself is a risk factor of major depression (Herpertz, 2015).

Furthermore, increased levels of perceived acute and chronic stress along with dysregulations of the endocrine stress response likely contribute to the genesis and maintenance of obesity (Morris et al., 2015). Chronic stress is biologically reflected by enduring stimulation of the hypothalamic-pituitary-adrenal (HPA) axis leading to elevated levels of circulating glucocorticoids (Strack et al., 1995; Morris et al., 2015). Subsequent catabolic effects and the impact on energy balance in terms of orexigenic signaling (e.g., via neuropeptide Y, NPY) further contribute to the maintenance of obesity and its comorbidities (Strack et al., 1995; Chrousos, 2009). Moreover, a pro-inflammatory state due to large amounts of endocrinely active visceral fat tissue, which is another stimulus of the HPA axis, and increased stress levels are in close interaction (Nezi et al., 2000; Tilg and Moschen, 2006). An important connection of this psychobiological relationship is the bidirectional gut-brain axis (Lee and Abizaid, 2014). In this crosstalk, intestinally and centrally secreted peptides are crucial (Holzer and Farzi, 2014; Lach et al., 2018). Long-term regulation of energy balance and body weight is redundantly regulated by several important hormones, e.g., insulin, leptin, and ghrelin (Pape, 2014). Therefore, dysregulation of peptides of the gut-brain axis are in the focus of obesity research (Beck, 2000; Boughton and Murphy, 2013). Nevertheless, several potentially relevant peptides have not yet been investigated in this context.

Peptides of the highly conserved neurotensin/xenopsin/xenin family exert diverse peripheral and central functions along the gut-brain axis (Feurle, 1998). A central anorexigenic effect by neurotensin and xenin was shown in rats, whereas, this effect has not been shown yet for the amphibian peptide xenopsin (Araki et al., 1975; Luttinger et al., 1982; Alexiou et al., 1998; McConn et al., 2015).

Neurotensin, a tridecapeptide, was detected in bovine hypothalamus (Carraway and Leeman, 1973). It is derived from the same precursor protein as neuromedin N (NMN), secreted in the central nervous system (CNS) and peripherally by N-cells of the intestinal mucosa due to postprandially increased free fatty acids (Kislauskis et al., 1988; Malendowicz, 1998; Schroeder and Leinninger, 2018). Important brain areas of neurotensin secretion are amygdala, hippocampus, ventral tegmental area (VTA), arcuate, and paraventricular nuclei (St-Gelais et al., 2006). Besides the anti-hypertensive effect of the peptide, many other effects were discovered (Blackburn, 1978) encompassing a stimulation of gut motility and pancreatic secretion of glucagon and insulin, a reduction of gastric acid secretion and a role as growth factor (Blackburn et al., 1980; Yawata et al., 1984; Carraway and Plona, 2006; Kalafatakis and Triantafyllou, 2011). Central effects of neurotensin are hypothermia, food intake suppression, and opioid-independent analgesia as well as an endogenous antipsychotic activity because of interactions with the dopaminergic system (Martin et al., 1980; Luttinger et al., 1982; Furuta et al., 1984; Fuxe et al., 1992). Furthermore, neurotensin displays regulatory functions in pituitary secretion of luteinizing hormone (LH) and follicle-stimulating hormone (FSH) (Herbison and Theodosis, 1991, 1992). Four different receptors are responsible for the physiological effects of neurotensin, especially the high-affinity neurotensin-receptor-1 (NTS_1_) and low-affinity neurotensin-receptor-2 (NTS_2_), which are G-protein-coupled-receptors (GPCRs) (Tanaka et al., 1990; Chalon et al., 1996; Mazella et al., 1998; Jacobsen et al., 2001).

Xenin, was discovered in 1992 but is still relatively little studied (Feurle et al., 1992). The 25 amino acid peptide hormone is released by enteroendocrine K-cells of the duodenum (Anlauf et al., 2000). The same cells synthesize glucose-dependent insulinotropic peptide (GIP) whose functions are closely related to the peripheral effects of xenin (Anlauf et al., 2000; Cho and Kieffer, 2010). Despite identical cells of origin in the duodenum, no sequence homology exists between the 42-amino acid polypeptide GIP and xenin (Feurle, 1998; Yip and Wolfe, 2000). GIP binds to the glucose-inhibitory peptide receptor, whereas, xenin primarily acts via the NTS_1_ (Clemens et al., 1997; Baggio and Drucker, 2007; Kim et al., 2016).

As observed for neurotensin, peripheral xenin is primarily released postprandially inducing a delayed gastric emptying rate, contraction of gall bladder, lipolysis of white adipose tissue, and stimulation of exocrine and endocrine pancreatic functions associated with an antidiabetic effect (Feurle et al., 1992; Kamiyama et al., 2007; Kim and Mizuno, 2010a; Wice et al., 2012; Bhavya et al., 2018). In the CNS, xenin suppresses appetite through effects on several food intake-regulatory centers in the hypothalamus and brainstem independent of melanocortin and leptin signaling pathways (Nandar et al., 2008; Leckstrom et al., 2009; Kim and Mizuno, 2010a). Xenin and neurotensin show 20% sequence homology (five amino acids in identical position), especially near the C-terminus (Yip and Wolfe, 2000).

Due to their physiological effects, both neurotensin and xenin have been examined in the context of obesity and in relation to somatic sequelae of the disease (Craig et al., 2018; Schroeder and Leinninger, 2018). Antidiabetic properties of xenin and dysregulations of neurotensin and pro-neurotensin in obesity and high-fat diet are current areas of interest (Weiss et al., 2001; Wice et al., 2010; Gault et al., 2015; Li et al., 2016; Hasib et al., 2017). Moreover, anorexigenic effects of both peptides have been examined, because in animal models of obesity, decreased central concentrations of neurotensin have been detected together with reduced food intake after intracerebroventricular (icv) administration of xenin and neurotensin in rodents (Beck et al., 1990; Williams et al., 1991a; Cooke et al., 2009). Until now, mental conditions of obese patients and xenin levels have not been investigated.

The aim of the present study was to investigate potential associations between plasma levels of neurotensin and xenin and perceived stress, anxiety, depressiveness, and eating disorder symptoms in obese inpatients. Women and men were examined separately since expression of neurotensin and were shown to be sex-dependent (Herbison and Theodosis, 1991, 1992; Alexander, 1993).

## Materials and Methods

### Participants

Investigations were conducted according to the Declaration of Helsinki. All participants gave written informed consent and the study was approved by the institutional ethics committee of the Charité – Universitätsmedizin Berlin (protocol number: EA1/130/16).

In the period between September 2010 and December 2016 we consecutively recruited 160 obese inpatients (97 women and 63 men). All participants were hospitalized in the Department of Psychosomatic Medicine Charité – Universitätsmedizin Berlin, where they received therapy due to obesity and its mental and somatic comorbidities. Inclusion criteria encompassed a BMI ≥ 30 kg/m^2^ and a minimum age of 18 years. Current pregnancy or lactation period, dialysis, hypercortisolism, malignoma, psychotic disorders as well as treatment with immunomodulatory drugs (e.g., methotrexate, azathioprine, and oral corticosteroids) were exclusion criteria. Furthermore, patients with somatic or somatoform disorders of the gastrointestinal tract or after surgery of the gastrointestinal tract including bariatric operations, except for appendectomy, and uncomplicated cholecystectomy, were excluded.

### Procedure

Study enrolment including clarification of potential exclusion criteria and blood withdrawal was conducted within four days after admission. Venous blood sample from a forearm vein of overnight fasted patients was taken every Friday between 7.00 am and 8.00 am. It was permitted to drink a small amount of water, but patients were not allowed to drink coffee or exercise before blood collection. Patients were also advised not to smoke prior to blood withdrawal. At the same morning we assessed actual medication, body height and fasting weight of participants in light underwear to calculate the BMI.

### Psychometric Measurements

Associated psychometric data collection occurred no more than two days before or five days after blood withdrawal. The following self-reported questionnaires were completed by the participants.

The Perceived Stress Questionnaire (PSQ) was used to evaluate the severity of subjective stress perception (Levenstein et al., 1993) with the shortened 20-item version (PSQ-20) by Fliege et al. (2005). The questionnaire encompasses the subscales “worries,” “tension,” “joy” with relation to the stress response and “demands” regarding the perception of external stressors (Levenstein et al., 1993). Scores ranged from zero to 100. Crohnbach’s alpha for the current sample was 0.892, 0.904, 0.824, and 0.838 for the four subscales, respectively, and 0.872 for the total score.

To evaluate the levels of anxiety and depression, we used two subscales of the Patient Health Questionnaire (PHQ) (Spitzer et al., 1999). For the assessment of anxiety, we used the German version (Löwe et al., 2008) of the Generalized Anxiety Disorder-7 (GAD-7) scale with seven items (4-point Likert scale with a range from zero up to three) as an established and widely used tool (Spitzer et al., 2006). Crohnbach’s alpha for the current sample was 0.903. The PHQ-9 with total scores from zero to 27 was designed to measure the severity of depressive symptoms and consists of nine items (Spitzer et al., 1999). We employed the German version of the subscale (Löwe et al., 2002). Crohnbach’s alpha for the current sample was 0.894.

The severity of eating disorder symptoms was measured by the Eating Disorder Inventory (EDI), which consists of 64 items (Garner et al., 1983). EDI comprises eight subscales covering different aspects of eating disorders: “drive for thinness,” “bulimia,” “body dissatisfaction,” “ineffectiveness,” “perfectionism,” “interpersonal distrust,” “interoceptive awareness,” and “maturity fears.” In detail, the German version of the EDI-2, a revised version of the EDI with three additional subscales (which were not applied in the current study) was used. Scores range from zero to 100 (Garner, 1991; Thiel et al., 1997). Crohnbach’s alpha for the current sample was 0.795, 0.896, 0.856, 0.901, 0.748, 0.784, 0.872, and 0.690 for the eight subscales, respectively, and 0.809 for the total score.

### Laboratory Analysis

Venous blood samples were collected in chilled standard laboratory EDTA tubes containing aprotinin (1.2 Trypsin Inhibitory Unit per 1 ml blood; ICN Pharmaceuticals, Costa Mesa, CA, United States) as peptidase inhibitor and stored on ice immediately after withdrawal. EDTA tubes were then centrifuged at 4°C for 10 min at 3000 *g*. Blood plasma of each sample was separated and stored at −80°C. Neurotensin and xenin were measured by the use of enzyme-linked immunosorbent assays (ELISA, neurotensin: catalog # EK-048-03, xenin: catalog # EK-046-74, Phoenix Pharmaceuticals Inc., Burlingame, CA, United States, 0% cross reactivity between both analytes according to manufacturer’s information). For neurotensin, the intra-assay variability was 5.8% and for xenin 7.3%. Every measurement was conducted twice, whereof we calculated the mean value.

### Statistical Analysis

Kolmogorov-Smirnov test was used to determine the distribution of the data. Because all data were normally distributed, we employed *t*-tests and chi-square tests analyzing differences between two groups. Effect sizes were estimated by Cohen’s *d* (*t*-test), Phi-coefficient *φ* (chi-square test) or Cramér’s *V* (chi-square test). Correlations were assessed using Pearson’s analysis. The correlations and differences between groups were considered significant when *p* < 0.05. Due to the explorative character of the study no *p* adjustment has been performed. Multivariable linear regression was used to investigate the effect of age, gender, various comorbidities and medication. Data are expressed as mean ± standard derivation (SD) and statistical analyses were executed using SigmaStat 3.1 (Systat Software, San Jose, CA, United States) and IBM SPSS Statistics Version 26.0.0.0 (IBM corp, Armonk, NY, United States).

## Results

### Characterization of the Study Population

Anthropometric, endocrine and psychometric characteristics of the study population (*n* = 160) and a comparison between women and men are presented in Table 1. Mean BMI was 47.2 ± 9.5 kg/m^2^ (range: 31.8–75.1 kg/m^2^) and mean age 45.5 ± 13.7 years (range: 19–73 years). PSQ-20 data was missing for one man, whereas, EDI-2 data was lacking in one woman. Regarding ELISA analyses, one outlier for neurotensin and two outliers for xenin (all in women) were excluded from further statistical calculations. In the whole study population, the mean plasma neurotensin concentration was 0.8 ± 0.5 ng/ml (range: 0.1–3.6 ng/ml), mean plasma xenin was 0.9 ± 0.5 ng/ml (range: 0.3–2.9 ng/ml).

**TABLE 1:** Anthropometric, psychometric, and endocrine characteristics of study populations.

Parameter	Men (*n* = 63)	Women (*n* = 97)	*p*	*d*
Age (years)	47.5 ± 13.6 (19–73)	44.2 ± 13.5 (20–73)	0.131	0.246
BMI (kg/m^2^)	45.5 ± 8.2 (32–67)	48.4 ± 10.0 (33–75)	0.059	0.307
PSQ-20 total score	47.0 ± 20.8 (3–95)	58.2 ± 21.7 (2–98)	**0.002**	0.521
- Worries	42.6 ± 24.9 (0–100)	59.1 ± 27.2 (0–100)	**<0.001**	0.624
- Tension	50.2 ± 26.7 (0–100)	61.6 ± 27.1 (0–100)	**0.011**	0.420
- Joy	42.5 ± 22.9 (0–100)	37.7 ± 24.0 (0–100)	0.214	0.203
- Demands	37.5 ± 25.2 (0–100)	49.6 ± 23.9 (0–100)	**0.003**	0.493
GAD-7 total score	7.1 ± 5.3 (0–20)	9.7 ± 5.8 (0–21)	**0.004**	0.469
PHQ-9 total score	8.8 ± 5.9 (0–25)	11.6 ± 6.6 (1–26)	**0.008**	0.436
EDI-2 total score	39.7 ± 11.9 (19–72)	50.5 ± 12.8 (27–84)	**<0.001**	0.866
- Drive for thinness	50.1 ± 21.6 (9–97)	61.3 ± 20.0 (17–97)	**0.001**	0.541
- Bulimia	22.2 ± 19.3 (0–74)	29.6 ± 24.4 (0–97)	**0.046**	0.327
- Body dissatisfaction	71.5 ± 21.7 (33–100)	89.4 ± 13.7 (46–100)	**<0.001**	1.030
- Ineffectiveness	31.1 ± 18.2 (0–78)	46.2 ± 21.0 (6–90)	**<0.001**	0.751
- Perfectionism	38.6 ± 19.0 (7–87)	43.2 ± 20.4 (7–93)	0.155	0.232
- Interpersonal distrust	40.3 ± 17.8 (0–80)	47.4 ± 17.4 (9–89)	**0.013**	0.405
- Interoceptive awareness	26.4 ± 18.2 (0–82)	39.8 ± 18.3 (6–90)	**<0.001**	0.726
- Maturity fears	37.5 ± 17.0 (10–86)	42.7 ± 16.8 (8–100)	0.064	0.302
Neurotensin (ng/ml)	0.8 ± 0.3 (0.1–1.8)	0.8 ± 0.5 (0.2–3.6)	0.835	0.034
Xenin (ng/ml)	0.9 ± 0.4 (0.3–2.4)	1.0 ± 0.5 (0.3–2.9)	0.131	0.247

*Data are expressed as mean ± SD, the range is indicated in parentheses. Differences between the two groups were assessed using t-test. Significant p values are indicated in bold. Cohen‘s d is indicated as marker for the effect size. BMI, body mass index; EDI-2, Eating Disorder Inventory-2; GAD-7, Generalized Anxiety Disorder-7; PHQ-9, Patient Health Questionnaire-9; PSQ-20, Perceived Stress Questionnaire-20.*

Psychometric parameters showed significant differences between gender groups (Table 1). Women displayed higher values for all psychometric parameters, namely perceived stress (+24%), anxiety (+37%), depressiveness (+32%), and eating disorder symptoms (+27%). Female and male participants did not show statistical differences concerning socioeconomic characteristics (Table 2). As shown in Table 2, female obese patients more often suffered from binge-eating disorder (BED) than male patients, while men showed significantly more somatic comorbidities. Insulin was a significantly more frequent component of medication in men (Table 2). However, testing these comorbidities and medication as potential confounders with help of multivariable linear regression indicated no significant influence, neither on neurotensin nor on xenin (Supplementary Table 1).

**TABLE 2:** Demographic and socioeconomic characteristics, comorbidities and medication.

Parameter	Whole population (*n* = 160)	Women (*n* = 97)	Men (*n* = 63)	*p*	*φ / V*
**Socioeconomic characteristics**					
Living in a partnership	64 (40%)	41 (42.27%)	23 (36.51%)	0.467	−0.057
Level of education				0.246	0.184
University entrance diploma	35 (21.88%)	16 (16.49%)	19 (30.16%)		
Vocational diploma	8 (5%)	5 (5.15%)	3 (4.76%)		
Secondary education certificate	65 (40.63%)	44 (45.36%)	21 (33.33%)		
Basic school qualification	38 (23.75%)	22 (22.68%)	16 (25.4%)		
No school-leaving qualification	14 (8.75%)	10 (10.31%)	4 (6.35%)		
Currently employed	45 (28.13%)	26 (26.8%)	19 (30.16%)	0.645	0.036
Unemployment during past 5 years	71 (44.38%)	41 (42.27%)	30 (47.62%)	0.506	0.053
**Comorbidities**					
Bulimia Nervosa	2 (1.25%)	2 (2.06%)	0 (0%)	0.251	0.091
Binge-eating disorder	32 (20%)	27 (27.84%)	5 (7.94%)	**0.002**	0.243
Sleep-associated breathing disorder (women: *n* = 79; men: *n* = 56)	64 (47.41%)	28 (35.44%)	36 (64.29%)	**<0.001**	−0.258
Type 2 diabetes mellitus	52 (32.5%)	29 (29.9%)	23 (36.51%)	0.383	−0.069
Arterial hypertension	102 (63.75%)	52 (53.61%)	50 (79.37%)	**<0.001**	−0.262
Hypercholesterinemia	87 (54.38%)	45 (46.39%)	42 (66.6%)	**0.012**	−0.199
Hypertriglyceridemia	43 (26.88%)	20 (20.62%)	23 (36.51%)	**0.027**	−0.175
Hyperuricemia (women: *n* = 89; men: *n* = 61)	69 (46%)	38 (42.7%)	31 (50.82%)	0.327	−0.080
Fatty liver disease (women: *n* = 75; men: *n* = 43)	79 (66.95%)	46 (61.33%)	33 (76.74%)	0.087	−0.158
**Medication**					
Insulin	18 (11.25%)	7 (7.22%)	11 (17.46%)	**0.045**	−0.158
DPP-4 antagonists/GLP-1 analogs	10 (6.25%)	5 (5.15%)	5 (7.94%)	0.478	−0.056
Antidiabetics (other)	32 (20%)	18 (18.56%)	14 (22.22%)	0.571	−0.045
Proton-pump inhibitor	49 (30.63%)	27 (27.84%)	22 (34.92%)	0.342	−0.075
Non-steroidal anti-inflammatory drugs	50 (31.25%)	29 (29.90%)	21 (33.34%)	0.647	−0.036
Psychopharmacological treatment	59 (36.88%)	39 (40.21%)	20 (31.75%)	0.278	0.086
Neuroleptics	19 (11.88%)	12 (12.37%)	7 (11.11%)	0.810	0.019
SSRI/SNRI	39 (24.38%)	28 (28.87%)	11 (17.46%)	0.101	0.130
Tricyclic antidepressants	10 (6.25%)	7 (7.22%)	3 (4.76%)	0.531	0.050
Other antidepressants	9 (5.63%)	6 (6.19%)	3 (4.76%)	0.703	0.030
Tranquilizers, sedatives, hypnotics	2 (1.25%)	1 (1.03%)	1 (1.59%)	0.757	−0.024
Other psychopharmacological medication	10 (6.25%)	6 (6.19%)	4 (6.35%)	0.967	−0.003

*Statistical analysis: Data are expressed as mean ± standard deviation or absolute number with percentage in parentheses. Differences between two groups were assessed using the chi-square test. Significant differences are displayed in bold. Phi-coefficient (φ) or Cramér’s V (Level of Education) are indicated as markers for the effect size. BMI, body mass index; DPP-4, dipeptidyl peptidase-4 inhibitor; GLP-1, glucagon-like peptide-1; SNRI, serotonin-norepinephrine reuptake inhibitor; SSRI, selective serotonin reuptake inhibitor.*

Patients with diabetes mellitus type 2 did not show differences in concentrations of neurotensin and xenin compared to those without diabetes (*t*-test; data not shown). Likewise, the peptide levels were similar in patients taking antidiabetic medication like insulin, DPP-4 antagonists/GLP-1 analogs or other antidiabetics, and in those without (*t*-test; data not shown).

After we excluded three women with polycystic ovary syndrome (PCOS), no significant difference of xenin concentrations was detectable (*p* = 0.900; *d* = 0.018).

Furthermore, there was no significant difference in plasma peptide levels between patients taking proton pump inhibitors (PPIs) or non-steroidal anti-inflammatory drugs (NSAIDs) and those not (*t*-test; data not shown). Fatty liver disease did not demonstrate a significant impact on neurotensin and xenin (*t*-test; data not shown). Also, when using linear regression for testing, we did not observe a significant predictive power of PPI’s, NSAID’s or of the diagnosis fatty liver disease, neither on neurotensin, nor xenin (Supplementary Table 2).

We also conducted multivariable linear regression analyses in order to estimate the influence of age and gender on xenin and neurotensin (xenin: *R*^2^_*cor*_ = 0.002; *p* = 0.319; neurotensin: *R*^2^_*cor*_ = −0.012; *p* = 0.968). Additionally, multivariable linear regression analyses for age and perceived stress, anxiety, depressiveness and eating disorder pathology, respectively, in women and men were calculated (Supplementary Table 1).

### Sex-Specific Associations Between Neurotensin and BMI, Age, and Psychometric Parameters

In men but not in women, a significant weak negative correlation was observed between BMI and neurotensin plasma levels (Table 3). Strong positive correlations were observed between neurotensin and xenin in women and men (Tables 3, 4).

**TABLE 3:** Correlation of neurotensin with anthropometric, psychometric and endocrine parameters.

Parameter	Men (*n* = 63)		Women (*n* = 97)	
	*r*	*p*	*r*	*p*
Age (years)	0.113	0.378	−0.028	0.787
BMI (kg/m^2^)	−0.265	**0.036**	−0.100	0.334
PSQ-20 total score	0.137	0.289	0.231	**0.023**
- Worries	−0.037	0.777	0.188	0.066
- Tension	0.145	0.262	0.274	**0.007**
- Joy	−0.142	0.271	−0.204	**0.046**
- Demands	0.208	0.105	0.113	0.273
GAD-7 total score	0.027	0.833	0.265	**0.009**
PHQ-9 total score	0.052	0.687	0.281	**0.006**
EDI-2 total score	−0.200	0.115	0.276	**0.007**
- Drive for thinness	−0.180	0.157	0.212	**0.039**
- Bulimia	−0.192	0.132	0.172	0.096
- Body dissatisfaction	−0.237	0.062	0.039	0.706
- Ineffectiveness	−0.065	0.613	0.229	**0.026**
- Perfectionism	0.046	0.719	0.336	**0.001**
- Interpersonal distrust	−0.117	0.360	0.201	0.051
- Interoceptive awareness	−0.064	0.620	0.252	**0.014**
- Maturity fears	−0.149	0.244	0.027	0.796
Xenin (ng/ml)	0.731	**<0.001**	0.788	**<0.001**

*Correlations were assessed using Pearson’s analysis. Significant correlations are indicated in bold. BMI, body mass index; EDI-2, Eating Disorder Inventory-2; GAD-7, Generalized Anxiety Disorder-7; PHQ-9, Patient Health Questionnaire-9; PSQ-20, Perceived Stress Questionnaire-20.*

**TABLE 4:** Correlation of xenin with anthropometric, psychometric and endocrine parameters.

Parameter	Men (*n* = 63)		Women (*n* = 97)	
	*r*	*p*	*r*	*p*
Age (years)	0.115	0.368	−0.041	0.696
BMI (kg/m^2^)	−0.089	0.487	0.045	0.664
PSQ-20 total score	−0.040	0.760	0.254	**0.013**
- Worries	−0.146	0.257	0.205	**0.046**
- Tension	0.020	0.878	0.267	**0.009**
- Joy	0.042	0.745	−0.230	**0.025**
- Demands	0.031	0.812	0.158	0.127
GAD-7 total score	−0.088	0.495	0.257	**0.012**
PHQ-9 total score	−0.092	0.472	0.241	**0.019**
EDI-2 total score	−0.152	0.233	0.260	**0.011**
- Drive for thinness	0.021	0.868	0.274	**0.008**
- Bulimia	−0.142	0.265	0.096	0.359
- Body dissatisfaction	−0.168	0.189	0.177	0.088
- Ineffectiveness	−0.138	0.282	0.242	**0.019**
- Perfectionism	0.158	0.215	0.407	**<0.001**
-Interpersonal distrust	−0.169	0.185	0.140	0.178
- Interoceptive awareness	−0.133	0.298	0.167	0.107
- Maturity fears	−0.108	0.398	−0.076	0.468
Neurotensin (ng/ml)	0.731	**<0.001**	0.788	**<0.001**

*Correlations were assessed using Pearson’s analysis. Significant correlations are indicated in bold. BMI, body mass index; EDI-2, Eating Disorder Inventory-2; GAD-7, Generalized Anxiety Disorder-7; PHQ-9, Patient Health Questionnaire-9; PSQ-20, Perceived Stress Questionnaire-20.*

**FIGURE 1:**
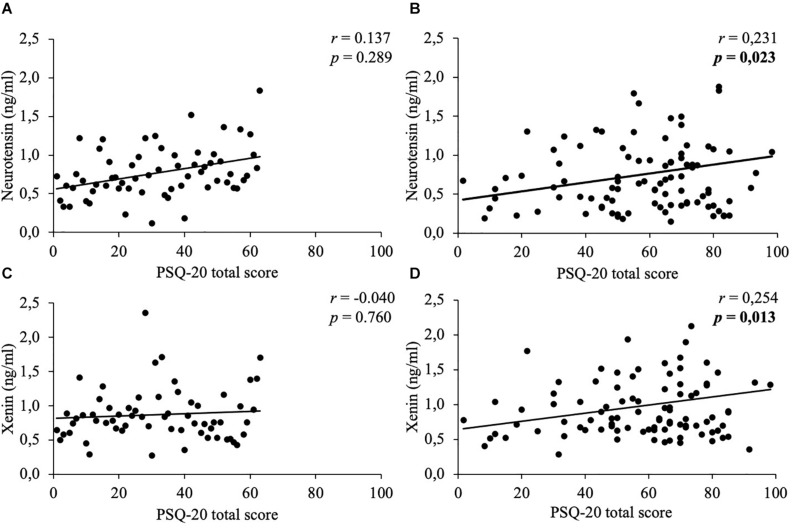
Correlations between neurotensin and xenin with PSQ-20 total scores in men and women. Overall, in women neurotensin **(B)** and xenin **(D)** showed a positive correlation with PSQ-20 total score. However, for men no significant correlations with PSQ-20 total score were detected, neither for neurotensin **(A)** nor for xenin **(C)**. Correlations were calculated using Pearson’s analysis. PSQ-20, Perceived Stress Questionnaire-20.

**FIGURE 2:**
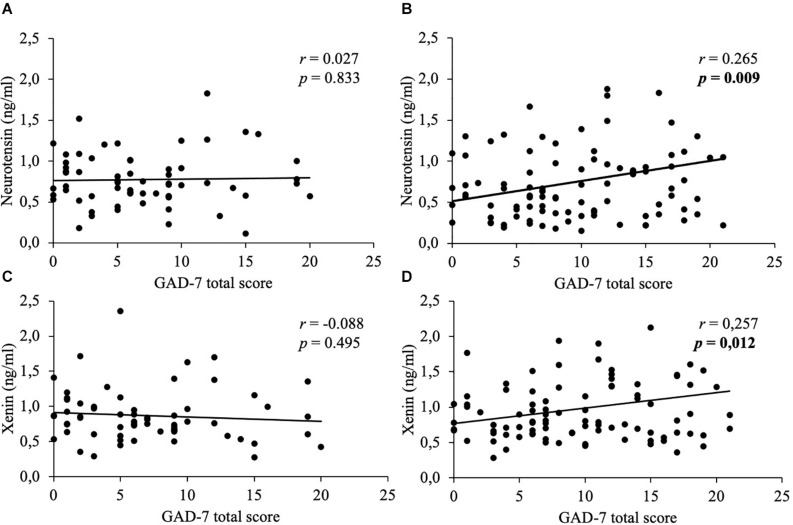
Correlations between neurotensin and xenin with GAD-7 total scores in men and women. Overall, in women neurotensin **(B)** and xenin **(D)** showed a positive correlation with GAD-7 total score, while for men no significant correlations with GAD-7 total score were detected, neither for neurotensin **(A)** nor for xenin **(C)**. Correlations were calculated using Pearson’s analysis. GAD-7, Generalized Anxiety Disorder-7.

**FIGURE 3:**
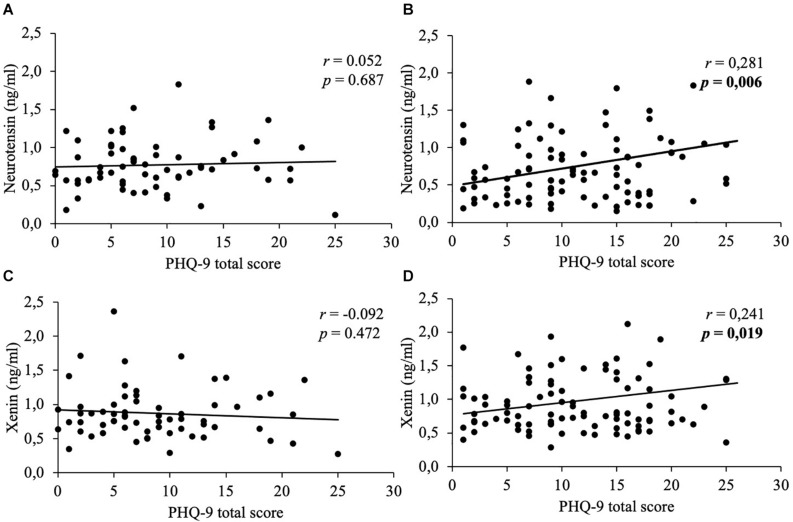
Correlations between neurotensin and xenin with PHQ-9 total scores in men and women. Overall, in women neurotensin **(B)** and xenin **(D)** showed a positive correlation with PHQ-9 total score. However, for men no significant correlations with PHQ-9 total score were detected, neither for neurotensin **(A)** nor for xenin **(C)**. Correlations were calculated using Pearson’s analysis. PHQ-9, Patient Health Questionnaire-9.

**FIGURE 4:**
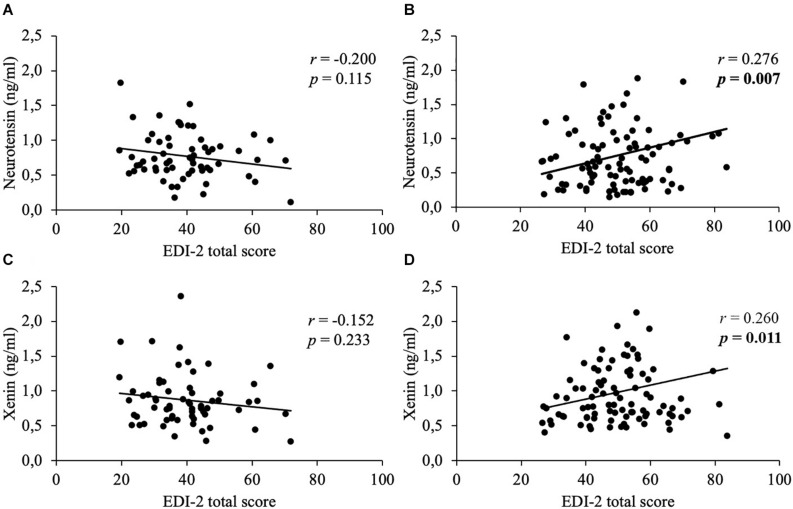
Correlations between neurotensin and xenin with EDI-2 total scores in men and women. Overall, in women neurotensin **(B)** and xenin **(D)** showed a positive correlation with EDI-2 total score, while for men no significant correlations with EDI-2 total score were detected, neither for neurotensin **(A)** nor for xenin **(C)**. Correlations were calculated using Pearson’s analysis. EDI-2, Eating Disorder Inventory-2.

In women, weak positive correlations between neurotensin and the total scores of the PSQ-20 (Figure 1), GAD-7 (Figure 2), PHQ-9 (Figure 3), and EDI-2 (Figure 4) were observed, while no such associations were observed in men (Table 3). However, reflecting weak positive correlations (Table 3), no significant association in multivariable linear regression for the independent variables of psychometrics (PSQ-20 total score; GAD-7; PHQ-9 and EDI-2 total score) and neurotensin was detectable in women (*R*^2^_*cor*_ = 0.052; *p* = 0.066).

### Sex-Specific Associations Between Xenin and BMI, Age, and Psychometrics

Weak positive associations of xenin and the total scores of all psychometric questionnaires were shown in obese women: PSQ-20 (Figure 1), GAD-7 (Figure 2), PHQ-9 (Figure 3), and EDI-2 (Figure 4 and Table 4). Due to the weakness of correlations (Table 4), no significant association in multivariable linear regression between the independent variables of psychometrics (PSQ-20 total score; GAD-7; PHQ-9 and EDI-2 total score) and xenin was detectable in women (*R*^2^_*cor*_ = 0.036; *p* = 0.123). In men, no significant correlations of the total and sub scores of applied psychometrics with xenin were detected (Table 4).

## Discussion

The aim of the present study was to examine potential associations between neurotensin and xenin in plasma with psychometrically assessed perceived stress, anxiety, depressiveness and eating disorder symptoms in patients with obesity. Positive correlations of neurotensin and xenin with the above-mentioned psychometric scores were only detectable in obese women, whereas, no correlations were observed in obese men.

Mood and anxiety disorders are more prevalent in obese compared to normal weight subjects (Baumeister and Härter, 2007; Herpertz, 2015). A current study (*n* = 2,955) demonstrated increased adjusted odds ratios (OR) for obese participants of the German National Health Interview and Examination Survey – Mental Health Supplement at a twelve-months follow up for mood and anxiety disorders (Jacobi et al., 2004; Baumeister and Härter, 2007). Especially obese women suffer more often than obese men from mental comorbidities like anxiety and mood disorders and pathologic eating behavior (Gater et al., 1998; Preiss et al., 2013; McHenry et al., 2014; Herpertz, 2015; Tronieri et al., 2017). This observation is in line with our current data showing higher values of determined psychological characteristics in women.

Detected sex-differences might be additionally influenced by variations in obesity-related stigmatization between women and men. The stigmatization appears in all facets of everyday life, verbal and non-verbal, with direct consequences regarding for example eating habits (Faith et al., 2002; Schwartz et al., 2003; Puhl and Brownell, 2006). In previous literature, differences regarding stigmatization exist between obese women and men, whereby women feel more often affected (Puhl et al., 2008). Especially middle-aged and morbidly obese females report great psychological distress (Puhl et al., 2008). This current stage of research is in line with our finding of higher psychometric values in obese females. Moreover, the risk for the development of BED seems to be associated with a high level of perceived weight-related stigmatization (Almeida et al., 2011), which is reflected by our result of significantly more BED diagnoses among women. Potential influencing factors in this context could be the increased emotional awareness in addition to the cognitive skill of women to interpret facial expressions of emotions better than men, as shown in several studies (Hall and Matsumoto, 2004; Mankus et al., 2016). Higher values of our female obese patients in the EDI-2 subscale “interoceptive awareness” is a supportive indicator for this influence.

Studies on various animal models showed decreased central concentrations of neurotensin under conditions of obesity (Williams et al., 1991b; Beck et al., 1992). In the periphery, controversial data have been obtained: decreased peripheral concentrations of neurotensin were detected in morbidly obese patients (Weiss et al., 2001), while another study described a higher risk for high fat diet-induced obesity in mice with higher concentrations of peripheral pro-neurotensin (Li et al., 2016) possibly reflecting species differences. Decreased concentrations of xenin in plasma of obese children were observed, whereas, obese adolescents showed increased serum levels (Mrozek et al., 2012; Arslan et al., 2014) pointing toward confounding factors such as comorbid psychiatric diseases influencing this association.

In the current study a negative association between neurotensin and BMI of obese men has been detected, which is in line with a previous study (Weiss et al., 2001), whereas, in other studies no difference in neurotensin levels between obese and lean subjects has been detected (Service et al., 1986; Auguet et al., 2018). However, these previous examinations bear several methodical limitations (small sample size) (Service et al., 1986), and methodical differences (gender distribution) (Weiss et al., 2001; Auguet et al., 2018). Thus, the comparability between results of these studies is limited. We assumed a positive association between psychometric parameters and BMI because of an increased number of comorbidities and elevated psychological strain with higher BMI. Consequently, the negative correlation might conceal higher plasma levels of neurotensin related to higher scores of questionnaires in morbidly obese men. However, one has to note that only an obese spectrum of body weight was assessed in the present study.

In light of the receptor distribution of NTS_1_ in certain brain areas, a role of neurotensin and xenin in the regulation of stress and emotions can be assumed since NTS_1_ is highly expressed in the amygdala and limbic system (Alexander and Leeman, 1998; Fassio et al., 2000). Regarding neurotensin, both anxiogenic and anxiolytic effects have been demonstrated in rats after injection into the bed nucleus of stria terminalis (BNST; anxiogenic) or ventral pallidum (anxiolytic) (Ollmann et al., 2015; Normandeau et al., 2018; Li et al., 2020) indicating a site-dependent effect of the peptide. Mean concentrations of neurotensin and xenin did not differ between female and male obese patients in our study, while only women showed positive correlations of both peptides with the examined psychological parameters giving rise to a sex-dependent association. A sex-specific regulation of neurotensin has been suggested previously. The peptide hormone is considerably higher concentrated in the anteroventral periventricular nucleus in female rats, influences the release of gonadotropin-releasing-hormone and in turn is itself affected by menstrual cycle and estrogens (Alexander et al., 1991; Herbison and Theodosis, 1991, 1992; Alexander, 1993; Dungan Lemko et al., 2010). As a consequence, also dysregulated levels of reproductive hormones in morbidly obese men may have an influence on central neurotensin concentrations and furthermore on the sex-dependent associations with psychometrics (Hammoud et al., 2008). Xenin is less investigated regarding potential sex-dependent differences compared to neurotensin. A case-control study showed an increase of serum xenin in women with PCOS versus women without PCOS (Norman et al., 2007; Azziz et al., 2016; Guclu et al., 2019). However, co-morbid PCOS of our female obese inpatients (three women) did not contribute to divergences of the examined associations. Therefore, sex hormones should be additionally analyzed in future research.

Neurotensin and stress have been closely linked. Early on it was shown that icv injection of neurotensin stimulates the secretion of adrenocorticotropic hormone (ACTH) and subsequently the adrenal glands in rodents (Gudelsky et al., 1989). Recently, it was shown that chronic stress induces central neurotensin release in rats (Normandeau et al., 2018). Orexigenic signaling through increased HPA axis hormones is a further stimulus for obesity (Strack et al., 1995; Chrousos, 2009) and might counteract the anorexigenic effect of neurotensin in CNS. This may indicate that higher neurotensin levels contribute to the development and/or maintenance of perceived stress and anxiety. For xenin, we found positive correlations with perceived stress and anxiety in women with obesity. In a previous study, a linear correlation between peripherally measured and centrally xenin levels gave rise to a transport across the blood-brain barrier (BBB) (van de Sande-Lee et al., 2013). Accordingly, given the current controversial state of research, elevated xenin levels in the CNS of obese women together with agonistic effects of the peptide at NTS_1_ may be an indicator for anxiogenic and stress-inducing consequences (Steele et al., 2017; Normandeau et al., 2018; Li et al., 2020). However, clarification of the signaling pathways is necessary to obtain a more detailed picture.

In the current study, depressiveness was positively correlated with neurotensin and xenin in obese women only. In this context, it is to note that female obese patients more often show a comorbid major depression compared to men (Herpertz, 2015). Moreover, several items of the PHQ-9, such as “dysregulated appetite” and “low self-esteem,” overlap with facets of other psychometric questionnaires (e.g., EDI-2) (Garner, 1991; Thiel et al., 1997; Löwe et al., 2002). This might suggest that correlations refer at least partially to similar constructs. In addition, mood disorders are common comorbidities of eating disorders, in adolescence and also over the whole lifespan (Casper, 1998; Swanson et al., 2011). A recent study reported a potential association between plasma neurotensin and the number of lifetime suicide attempts in depressive patients, with the result of a strong positive correlation (Kim et al., 2019) further suggesting an involvement of neurotensin in the pathogenesis of depression.

Similarly, neurotensin and xenin were correlated with parameters of disordered eating in obese women only. This might be related to higher prevalence rates of eating disorders in women, especially for anorexia nervosa (AN) and for bulimia nervosa (BN), with up to tenfold higher prevalence in women (Arbeitsgemeinschaft Wissenschaftlicher Medizinischer Fachgesellschaften, 2018). BED was more prevalent in our female participants, which might be contributing to the lacking correlation in men. Furthermore, an association of sex hormones with disordered eating behavior and obesity is notable (Hirschberg, 2012). Testosterone levels are elevated in obese women following increased chronic stress and hyperinsulinemia, which promotes binge eating and abdominal fat distribution (Torres and Nowson, 2007; Hirschberg, 2009, 2012). Moreover, the HPA axis is overstimulated through endocrine activity of the abdominal fat (Torres and Nowson, 2007; Pasquali et al., 2008; Hirschberg, 2009, 2012). In obese men, estradiol concentrations are elevated and the quantity of abdominal fat leads to lower testosterone concentrations (Hammoud et al., 2008; Pasquali et al., 2008; Hirschberg, 2012). Data concerning the relationship of xenin with depressiveness or disordered eating behavior are lacking, but due to same receptor and the high sequence homology, similar explanations as for neurotensin may be assumed (Feurle, 1998; Maes et al., 2001; Kim and Mizuno, 2010b; Li et al., 2020). Thus, future studies should focus on xenin.

Several limitations of the study should be kept in mind. First, we did not assess the menstrual status, and the intake of estrogen-containing medication was not considered as an exclusion criterion. Second, due to the naturalistic design no healthy control group was employed in our study. We therefore were not able to compare our findings in obese subjects with normal weight individuals without mental disorders under study conditions. However, the naturalistic design is also a strength of this examination because it reproduces real world conditions during psychosomatic inpatient treatment. Third, the cross-sectional study design observes associations, no cause-effect relationships. Hence, besides experimental studies featuring healthy control groups, future longitudinal studies are required assessing neurotensin and xenin levels in the course of psychopathology-improving therapies. Lastly, heterogeneous comorbidities are potentially representing confounding factors and therefore might contribute – besides the relatively small sample size – to the observed weak associations. Thus, further studies with bigger sample size or with more stratified study collectives are needed.

Based on previous literature, increased xenin levels following treatment with PPI’s as well as decreased plasma concentrations of neurotensin in women with fatty liver disease and obesity were described (Stoschus et al., 1998; Auguet et al., 2018). Previous studies indicated that gastrointestinal (initiation of gut motility and inhibition of gastric acid production) and central effects of neurotensin depend on prostaglandin (Mason et al., 1982; Katsoulis and Conlon, 1988), why we investigated a possible influence of the intake of NSAID’s on peptide levels. Until know, interactions between NSAID’s and xenin were not assessed, but imaginable (Stoschus et al., 1998; Kapraali et al., 1999). However, in our study NSAID’s did not affect peripheral levels of neurotensin or xenin.

Taken together, we observed positive correlations of neurotensin and xenin with stress, anxiety, depressiveness and eating disorder symptoms in obese women, whereas, associations were absent in male patients with obesity indicating a sex-specific association. Whether this association represents a causal relationship should be further investigated. If so, it would be interesting to investigate whether inhibition of these peptides could exert a beneficial effect on these psychometric parameters. Lastly, due to the sequence homology between neurotensin and xenin along with the – at least in part – similar activation of the NTS_1_ it will be interesting to investigate whether the peptides are be able to compensate each other.

## Data Availability Statement

The original contributions presented in the study are included in the article/Supplementary Material, further inquiries can be directed to the corresponding author/s.

## Ethics Statement

The studies involving human participants were reviewed and approved by Institutional Ethics Committee of the Charité – Universitätmedizin Berlin (protocol number: EA1/130/16). The patients/participants provided their written informed consent to participate in this study.

## Author Contributions

EW and SS collected the samples. EW analyzed the data and wrote the first draft of the manuscript. TH and AS designed the study. TH, MR, and AS gave critical input throughout the work. AS analyzed the data. All authors finalized and agreed on the final version of the manuscript.

## Conflict of Interest

The authors declare that the research was conducted in the absence of any commercial or financial relationships that could be construed as a potential conflict of interest.

## References

[B1] Alexander M. J. (1993). Estrogen-regulated synthesis of neurotensin in neurosecretory cells of the hypothalamic arcuate nucleus in the female rat. *Endocrinology* 133, 1809–1816. 10.1210/endo.133.4.8404623 8404623

[B2] Alexander M. J. Kiraly Z. J. Leeman S. E. (1991). Sexually dimorphic distribution of neurotensin/neuromedin N mRNA in the rat preoptic area. *J. Comp. Neurol.* 311 84–96. 10.1002/cne.903110107 1939736

[B3] Alexander M. J. Leeman S. E. (1998). Widespread expression in adult rat forebrain of mRNA encoding high-affinity neurotensin receptor. *J. Comp. Neurol.* 402 475–500. 10.1002/(SICI)1096-9861(19981228)402:4<475::AID-CNE4<3.0.CO;2-9 9862322

[B4] Alexiou C. Zimmermann J. P. Schick R. R. Schusdziarra V. (1998). Xenin–a novel suppressor of food intake in rats. *Brain Res.* 800 294–299. 10.1016/s0006-8993(98)00535-6 9685684

[B5] Almeida L. Savoy S. Boxer P. (2011). The role of weight stigmatization in cumulative risk for binge eating. *J. Clin. Psychol.* 67 278–292. 10.1002/jclp.20749 21254056

[B6] Anlauf M. Weihe E. Hartschuh W. Hamscher G. Feurle G. E. (2000). Localization of xenin-immunoreactive cells in the duodenal mucosa of humans and various mammals. *J. Histochem. Cytochem. Official J. Histochem. Soc.* 48 1617–1626. 10.1177/002215540004801205 11101630

[B7] Araki K. Tachibana S. Uchiyama M. Nakajima T. Yasuhara T. (1975). Isolation and structure of a new active peptide xenopsin on rat stomach strip and some biogenic amines in the skin of xenopus laevis. *Chem. Pharm. Bull.* 23 3132–3140. 10.1248/cpb.23.3132 1218451

[B8] Arbeitsgemeinschaft Wissenschaftlicher Medizinischer Fachgesellschaften (2018). *S3-Leitlinie Diagnostik und Behandlung der Essstörungen*. Available online at: https://www.awmf.org/uploads/tx_szleitlinien/051-026l_S3_Essstoerung-Diagnostik-Therapie_2020-03.pdf (accessed October 11, 2020).

[B9] Arslan N. Sayin O. Tokgoz Y. (2014). Evaluation of serum xenin and ghrelin levels and their relationship with nonalcoholic fatty liver disease and insulin resistance in obese adolescents. *J. Endocrinol. Invest.* 37 1091–1097. 10.1007/s40618-014-0160-z 25200997

[B10] Auguet T. Aragonés G. Berlanga A. Martínez S. Sabench F. Aguilar C. (2018). Low circulating levels of neurotensin in women with nonalcoholic fatty liver disease associated with severe obesity. *Obesity (Silver Spring)* 26 274–278. 10.1002/oby.22058 29276861

[B11] Azziz R. Carmina E. Chen Z. Dunaif A. Laven J. S. Legro R. S. (2016). Polycystic ovary syndrome. *Nat. Rev. Dis. Primers.* 2:16057. 10.1038/nrdp.2016.57 27510637

[B12] Baggio L. L. Drucker D. J. (2007). Biology of incretins: GLP-1 and GIP. *Gastroenterology* 132 2131–2157. 10.1053/j.gastro.2007.03.054 17498508

[B13] Baumeister H. Härter M. (2007). Mental disorders in patients with obesity in comparison with healthy probands. *Int. J. Obes. (Lond)* 31 1155–1164. 10.1038/sj.ijo.0803556 17264844

[B14] Beck B. (2000). Neuropeptides and obesity. *Nutrition* 16 916–923. 10.1016/s0899-9007(00)00410-x 11054597

[B15] Beck B. Burlet A. Nicolas J. P. Burlet C. (1990). Hyperphagia in obesity is associated with a central peptidergic dysregulation in rats. *J. Nutr.* 120 806–811. 10.1093/jn/120.7.806 2366113

[B16] Beck B. Stricker-Krongrad A. Burlet A. Nicolas J. P. Burlet C. (1992). Changes in hypothalamic neurotensin concentrations and food intake in rats fed a high fat diet. *Int. J. Obes. Related Metab. Dis. J. Int. Assoc. Study Obes.* 16 361–366. 1319971

[B17] Bhavya S. Lew P. S. Mizuno T. M. (2018). Stimulation of white adipose tissue lipolysis by xenin, a neurotensin-related peptide. *Biochem. Biophys. Res. Commun.* 498 842–848. 10.1016/j.bbrc.2018.03.067 29530527

[B18] Blackburn A. M. (1978). Neurotensin. *J. Clin. Pathol. Suppl. (Assoc. Clin. Pathol.)* 8 12–16. 10.1016/j.bbadis.2017.12.036 399607 PMC1436062

[B19] Blackburn A. M. Fletcher D. R. Bloom S. R. Christofides N. D. Long R. G. Fitzpatrick M. L. (1980). Effect of neurotensin on gastric function in man. *Lancet (London, England)* 1 987–989. 10.1016/s0140-6736(80)91434-8 6103384

[B20] Boughton C. K. Murphy K. G. (2013). Can neuropeptides treat obesity? A review of neuropeptides and their potential role in the treatment of obesity. *Br. J. Pharmacol.* 170 1333–1348. 10.1111/bph.12037 23121386 PMC3838680

[B21] Carraway R. Leeman S. E. (1973). The isolation of a new hypotensive peptide, neurotensin, from bovine hypothalami. *J. Biol. Chem.* 248 6854–6861. 10.1016/S0021-9258(19)43429-7 4745447

[B22] Carraway R. E. Plona A. M. (2006). Involvement of neurotensin in cancer growth: evidence, mechanisms and development of diagnostic tools. *Peptides* 27 2445–2460. 10.1016/j.peptides.2006.04.030 16887236

[B23] Casper R. C. (1998). Depression and eating disorders. *Depress. Anxiety* 8(Suppl. 1) 96–104. 10.1002/(SICI)1520-6394(1998)8:1+<96::AID-DA15>3.0.CO;2-4 9809221

[B24] Chalon P. Vita N. Kaghad M. Guillemot M. Bonnin J. Delpech B. (1996). Molecular cloning of a levocabastine-sensitive neurotensin binding site. *FEBS Lett.* 386 91–94. 10.1016/0014-5793(96)00397-3 8647296

[B25] Cho Y. M. Kieffer T. J. (2010). K-cells and glucose-dependent insulinotropic polypeptide in health and disease. *Vitam. Horm.* 84 111–150. 10.1016/b978-0-12-381517-0.00004-7 21094898

[B26] Chrousos G. P. (2009). Stress and disorders of the stress system. *Nat. Rev. Endocrinol.* 5 374–381. 10.1038/nrendo.2009.106 19488073

[B27] Clemens A. Katsoulis S. Nustede R. Seebeck J. Seyfarth K. Morys-Wortmann C. (1997). Relaxant effect of xenin on rat ileum is mediated by apamin-sensitive neurotensin-type receptors. *Am. J. Physiol.* 272(1 Pt 1), G190–G196. 10.1152/ajpgi.1997.272.1.G190 9038893

[B28] Cooke J. H. Patterson M. Patel S. R. Smith K. L. Ghatei M. A. Bloom S. R. (2009). Peripheral and central administration of xenin and neurotensin suppress food intake in rodents. *Obesity (Silver Spring)* 17 1135–1143. 10.1038/oby.2008.652 19214175

[B29] Craig S. L. Gault V. A. Irwin N. (2018). Emerging therapeutic potential for xenin and related peptides in obesity and diabetes. *Diabetes Metab. Res. Rev.* 34:e3006. 10.1002/dmrr.3006 29633491

[B30] Dungan Lemko H. M. Naderi R. Adjan V. Jennes L. H. Navarro V. M. Clifton D. K. (2010). Interactions between neurotensin and GnRH neurons in the positive feedback control of GnRH/LH secretion in the mouse. *Am. J. Physiol. Endocrinol. Metab.* 298 E80–E88. 10.1152/ajpendo.00380.2009 19861584 PMC2806107

[B31] Faith M. S. Leone M. A. Ayers T. S. Heo M. Pietrobelli A. (2002). Weight criticism during physical activity, coping skills, and reported physical activity in children. *Pediatrics* 110(2 Pt 1):e23. 10.1542/peds.110.2.e23 12165622

[B32] Fassio A. Evans G. Grisshammer R. Bolam J. P. Mimmack M. Emson P. C. (2000). Distribution of the neurotensin receptor NTS1 in the rat CNS studied using an amino-terminal directed antibody. *Neuropharmacology* 39 1430–1442. 10.1016/s0028-3908(00)00060-5 10818259

[B33] Feurle G. E. (1998). Xenin–a review. *Peptides* 19 609–615. 10.1016/s0196-9781(97)00378-1 9533652

[B34] Feurle G. E. Hamscher G. Kusiek R. Meyer H. E. Metzger J. W. (1992). Identification of xenin, a xenopsin-related peptide, in the human gastric mucosa and its effect on exocrine pancreatic secretion. *J. Biol. Chem.* 267 22305–22309. 1429581

[B35] Fliege H. Rose M. Arck P. Walter O. B. Kocalevent R. D. Weber C. (2005). The Perceived Stress Questionnaire (PSQ) reconsidered: validation and reference values from different clinical and healthy adult samples. *Psychosom. Med.* 67 78–88. 10.1097/01.psy.0000151491.80178.78 15673628

[B36] Furuta S. Kisara K. Sakurada S. Sakurada T. Sasaki Y. Suzuki K. (1984). Structure-antinociceptive activity studies with neurotensin. *Br. J. Pharmacol.* 83 43–48. 10.1111/j.1476-5381.1984.tb10117.x 6435708 PMC1987165

[B37] Fuxe K. Von Euler G. Agnati L. F. Merlo Pich E. O’Connor W. T. Tanganelli S. (1992). Intramembrane interactions between neurotensin receptors and dopamine D2 receptors as a major mechanism for the neuroleptic-like action of neurotensin. *Annal. N. Y. Acad. Sci.* 668 186–204. 10.1111/j.1749-6632.1992.tb27350.x 1361113

[B38] Garg S. K. Maurer H. Reed K. Selagamsetty R. (2014). Diabetes and cancer: two diseases with obesity as a common risk factor. *Diabetes Obes. Metab.* 16 97–110. 10.1111/dom.12124 23668396 PMC3904746

[B39] Garner D. M. (1991). *EDI-2. Eating-Disorder-Inventory-2. Professional Manual*. Odessa. FL: Psychological Assessment Resources.

[B40] Garner D. M. Olmstead M. P. Polivy J. (1983). Development and validation of a multidimensional eating disorder inventory for anorexia nervosa and bulimia. *Int. J. Eat. Disord.* 2 15–34. 10.1002/1098-108X(198321)2:2<15::AID-EAT2260020203<3.0.CO;2-6

[B41] Gater R. Tansella M. Korten A. Tiemens B. G. Mavreas V. G. Olatawura M. O. (1998). Sex differences in the prevalence and detection of depressive and anxiety disorders in general health care settings: report from the World Health Organization Collaborative Study on Psychological Problems in General Health Care. *Arch. Gen. Psychiatry* 55 405–413. 10.1001/archpsyc.55.5.405 9596043

[B42] Gault V. A. Martin C. M. Flatt P. R. Parthsarathy V. Irwin N. (2015). Xenin-25[Lys13PAL]: a novel long-acting acylated analogue of xenin-25 with promising antidiabetic potential. *Acta Diabetol.* 52 461–471. 10.1007/s00592-014-0681-0 25374384

[B43] Grundy S. M. (2002). Obesity, metabolic syndrome, and coronary atherosclerosis. *Circulation* 105 2696–2698. 10.1161/01.cir.0000020650.86137.84 12057978

[B44] Guclu Y. A. Sahin E. Aksit M. (2019). The relationship between elevated serum xenin and insulin resistance in women with polycystic ovary syndrome: a case-control study. *Gynecol. Endocrinol.* 35 960–964. 10.1080/09513590.2019.1604663 31010340

[B45] Gudelsky G. A. Berry S. A. Meltzer H. Y. (1989). Neurotensin activates tuberoinfundibular dopamine neurons and increases serum corticosterone concentrations in the rat. *Neuroendocrinology* 49 604–609. 10.1159/000125176 2549437

[B46] Hall J. A. Matsumoto D. (2004). Gender differences in judgments of multiple emotions from facial expressions. *Emotion* 4 201–206. 10.1037/1528-3542.4.2.201 15222856

[B47] Hammoud A. O. Gibson M. Peterson C. M. Meikle A. W. Carrell D. T. (2008). Impact of male obesity on infertility: a critical review of the current literature. *Fertil. Steril.* 90 897–904. 10.1016/j.fertnstert.2008.08.026 18929048

[B48] Hasib A. Ng M. T. Gault V. A. Khan D. Parthsarathy V. Flatt P. R. (2017). An enzymatically stable GIP/xenin hybrid peptide restores GIP sensitivity, enhances beta cell function and improves glucose homeostasis in high-fat-fed mice. *Diabetologia* 60 541–552. 10.1007/s00125-016-4186-y 28004148 PMC6518372

[B49] Herbison A. E. Theodosis D. T. (1991). Neurotensin-lmmunoreactive neurons in the rat medial preoptic area are oestrogen-receptive. *J. Neuroendocrinol.* 3 587–589. 10.1111/j.1365-2826.1991.tb00322.x 19215527

[B50] Herbison A. E. Theodosis D. T. (1992). Localization of oestrogen receptors in preoptic neurons containing neurotensin but not tyrosine hydroxylase, cholecystokinin or luteinizing hormone-releasing hormone in the male and female rat. *Neuroscience* 50 283–298. 10.1016/0306-4522(92)90423-y 1359459

[B51] Herpertz S. (2015). “Adipositas und psychische Komorbidität,” in *Handbuch Essstörungen und Adipositas; Berlin, Heidelberg*, eds Herpertz S. Zwaan M. Zipfel S. (Berlin: Springer), 425–429. 10.1007/978-3-642-54573-3_54

[B52] Hirschberg A. L. (2009). Polycystic ovary syndrome, obesity and reproductive implications. *Womens Health (Lond)* 5 529–540. 10.2217/whe.09.39 19702452

[B53] Hirschberg A. L. (2012). Sex hormones, appetite and eating behaviour in women. *Maturitas* 71 248–256. 10.1016/j.maturitas.2011.12.016 22281161

[B54] Holzer P. Farzi A. (2014). Neuropeptides and the microbiota-gut-brain axis. *Adv. Exp. Med. Biol.* 817 195–219. 10.1007/978-1-4939-0897-4_9 24997035 PMC4359909

[B55] Jacobi F. Wittchen H. U. Holting C. Höfler M. Pfister H. Müller N. (2004). Prevalence, co-morbidity and correlates of mental disorders in the general population: results from the German Health Interview and Examination Survey (GHS). *Psychol. Med.* 34 597–611. 10.1017/s0033291703001399 15099415

[B56] Jacobsen L. Madsen P. Jacobsen C. Nielsen M. S. Gliemann J. Petersen C. M. (2001). Activation and functional characterization of the mosaic receptor SorLA/LR11. *J. Biol. Chem.* 276 22788–22796. 10.1074/jbc.M100857200 11294867

[B57] Kalafatakis K. Triantafyllou K. (2011). Contribution of neurotensin in the immune and neuroendocrine modulation of normal and abnormal enteric function. *Regul. Peptides* 170 7–17. 10.1016/j.regpep.2011.04.005 21549161

[B58] Kamiyama Y. Aihara R. Nakabayashi T. Mochiki E. Asao T. Kuwano H. (2007). The peptide hormone xenin induces gallbladder contractions in conscious dogs. *Neurogastroenterol. Motil. Official J. Eur. Gastrointestinal Motil. Soc.* 19 233–240. 10.1111/j.1365-2982.2006.00881.x 17300294

[B59] Kapraali M. Johansson O. Uribe A. (1999). Endogenous prostaglandins are physiological regulators of endocrine cells in the gastroduodenal mucosa of the rat. *Regul. Pept.* 83 105–116. 10.1016/s0167-0115(99)00053-1 10511464

[B60] Katsoulis S. Conlon J. M. (1988). Neurotensin and prostaglandin interactions in smooth muscle of the guinea pig stomach. *Eur. J. Pharmacol.* 158 251–256. 10.1016/0014-2999(88)90074-x 2855314

[B61] Kim D. J. Blossom S. J. Delgado P. L. Carbajal J. M. Cáceda R. (2019). Examination of pain threshold and neuropeptides in patients with acute suicide risk. *Prog. Neuropsychopharmacol. Biol. Psychiatry* 95:109705. 10.1016/j.pnpbp.2019.109705 31326514 PMC7309511

[B62] Kim E. R. Lew P. S. Spirkina A. Mizuno T. M. (2016). Xenin-induced feeding suppression is not mediated through the activation of central extracellular signal-regulated kinase signaling in mice. *Behav. Brain Res.* 312 118–126. 10.1016/j.bbr.2016.06.026 27316340

[B63] Kim E. R. Mizuno T. M. (2010a). Xenin delays gastric emptying rate and activates the brainstem in mice. *Neurosci. Lett.* 481 59–63. 10.1016/j.neulet.2010.06.055 20599589

[B64] Kim E. R. Mizuno T. M. (2010b). Role of neurotensin receptor 1 in the regulation of food intake by neuromedins and neuromedin-related peptides. *Neurosci. Lett.* 468 64–67. 10.1016/j.neulet.2009.10.064 19857548

[B65] Kislauskis E. Bullock B. McNeil S. Dobner P. R. (1988). The rat gene encoding neurotensin and neuromedin N. Structure, tissue-specific expression, and evolution of exon sequences. *J. Biol. Chem.* 263 4963–4968. 10.1016/S0021-9258(18)68881-7 2832414

[B66] Lach G. Schellekens H. Dinan T. G. Cryan J. F. (2018). Anxiety, depression, and the microbiome: a role for gut peptides. *Neurotherapeutics* 15 36–59. 10.1007/s13311-017-0585-0 29134359 PMC5794698

[B67] Leckstrom A. Kim E. R. Wong D. Mizuno T. M. (2009). Xenin, a gastrointestinal peptide, regulates feeding independent of the melanocortin signaling pathway. *Diabetes* 58 87–94. 10.2337/db08-0260 18984739 PMC2606897

[B68] Lee C. Y. Abizaid A. (2014). The gut-brain-axis as a target to treat stress-induced obesity. *Front. Endocrinol. (Lausanne)* 5:117. 10.3389/fendo.2014.00117 25101055 PMC4102906

[B69] Levenstein S. Prantera C. Varvo V. Scribano M. L. Berto E. Luzi C. (1993). Development of the perceived stress questionnaire: a new tool for psychosomatic research. *J. Psychosom. Res.* 37 19–32. 10.1016/0022-3999(93)90120-5 8421257

[B70] Li B. Chang L. L. Xi K. (2020). Neurotensin 1 receptor in the prelimbic cortex regulates anxiety-like behavior in rats. *Prog. Neuropsychopharmacol. Biol. Psychiatry* 104:110011. 10.1016/j.pnpbp.2020.110011 32561375

[B71] Li J. Song J. Zaytseva Y. Y. Liu Y. Rychahou P. Jiang K. (2016). An obligatory role for neurotensin in high-fat-diet-induced obesity. *Nature* 533 411–415. 10.1038/nature17662 27193687 PMC5484414

[B72] Löwe B. Decker O. Müller S. Brähler E. Schellberg D. Herzog W. (2008). Validation and standardization of the generalized anxiety disorder screener (GAD-7) in the general population. *Med. Care* 46 266–274. 10.1097/MLR.0b013e318160d093 18388841

[B73] Löwe B. Spritzer R. Zipfel S. Herzog W. (2002). *Gesundheitsfragebogen für Patienten (PHQ-D). Manual und Testunterlagen (2002) 2. Auflage (PRIME MD Patient Health Questionaire (PHQ) - German version. Manual and materials*, 2nd Edn. (Karlsruhe: Pfizer GmbH).

[B74] Luttinger D. King R. A. Sheppard D. Strupp J. Nemeroff C. B. Prange A. J. Jr. (1982). The effect of neurotensin on food consumption in the rat. *Eur. J. Pharmacol.* 81 499–503. 10.1016/0014-2999(82)90116-9 6811292

[B75] Maes M. Monteleone P. Bencivenga R. Goossens F. Maj M. van West D. (2001). Lower serum activity of prolyl endopeptidase in anorexia and bulimia nervosa. *Psychoneuroendocrinology* 26 17–26. 10.1016/s0306-4530(00)00032-9 11070331

[B76] Malendowicz L. K. (1998). Role of neuromedins in the regulation of adrenocortical function. *Hormone Metab. Res.* 30 374–383. 10.1055/s-2007-978901 9694566

[B77] Mankus A. M. Boden M. T. Thompson R. J. (2016). Sources of variation in emotional awareness: age, gender, and socioeconomic status. *Pers. Individ. Dif.* 89 28–33. 10.1016/j.paid.2015.09.043 26500384 PMC4612349

[B78] Martin G. E. Bacino C. B. Papp N. L. (1980). Hypothermia elicited by the intracerebral microinjection of neurotensin. *Peptides* 1 333–339. 10.1016/0196-9781(80)90011-x 7301636

[B79] Mason G. A. Hernandez D. E. Nemeroff C. B. Adcock J. W. Hatley O. L. Prange A. J. Jr. (1982). Interaction of neurotensin with prostaglandin E2 and prostaglandin synthesis inhibitors: effects on colonic temperature in mice. *Regul. Pept.* 4 285–292. 10.1016/0167-0115(82)90121-5 6960393

[B80] Mazella J. Zsurger N. Navarro V. Chabry J. Kaghad M. Caput D. (1998). The 100-kDa neurotensin receptor is gp95/sortilin, a non-G-protein-coupled receptor. *J. Biol. Chem.* 273 26273–26276. 10.1074/jbc.273.41.26273 9756851

[B81] McConn B. R. Park J. Gilbert E. R. Cline M. A. (2015). A novel role for xenopsin: stimulation of food intake. *Behav. Brain Res.* 292 500–507. 10.1016/j.bbr.2015.06.039 26151286

[B82] McHenry J. Carrier N. Hull E. Kabbaj M. (2014). Sex differences in anxiety and depression: role of testosterone. *Front. Neuroendocrinol.* 35:42–57. 10.1016/j.yfrne.2013.09.001 24076484 PMC3946856

[B83] Morris M. J. Beilharz J. E. Maniam J. Reichelt A. C. Westbrook R. F. (2015). Why is obesity such a problem in the 21st century? The intersection of palatable food, cues and reward pathways, stress, and cognition. *Neurosci. Biobehav. Rev.* 58 36–45. 10.1016/j.neubiorev.2014.12.002 25496905

[B84] Mrozek B. Tomasik P. J. Wedrychowicz A. Wojcik M. Skoczen S. Fyderek K. (2012). Plasma xenin concentrations in children. *Pediatric. Endocrinol. Diab. Metabol.* 18 5–8. 22525684

[B85] Nandar W. Milligan J. M. Cline M. A. (2008). Mechanisms of xenin-induced anorectic response in chicks (Gallus gallus). *Gen. Comp. Endocrinol.* 157 58–62. 10.1016/j.ygcen.2008.03.012 18440536

[B86] Nezi M. Mastorakos G. Mouslech Z. (2000). “Corticotropin releasing hormone and the immune/inflammatory response,” in *Endotext*, eds Feingold K. R. Anawalt B. Boyce A. Chrousos G. de Herder W. W. Dungan K. (South Dartmouth, MA: MDText.com, Inc). 25905246

[B87] Norman R. J. Dewailly D. Legro R. S. Hickey T. E. (2007). Polycystic ovary syndrome. *Lancet* 370 685–697. 10.1016/s0140-6736(07)61345-2 17720020

[B88] Normandeau C. P. Ventura-Silva A. P. Hawken E. R. Angelis S. Sjaarda C. Liu X. (2018). A key role for neurotensin in chronic-stress-induced anxiety-like behavior in rats. *Neuropsychopharmacol. Official Publication Am. Coll. Neuropsychopharmacol.* 43 285–293. 10.1038/npp.2017.134 28649992 PMC5729557

[B89] Ollmann T. Peczely L. Laszlo K. Kovacs A. Galosi R. Kertes E. (2015). Anxiolytic effect of neurotensin microinjection into the ventral pallidum. *Behav. Brain Res.* 294 208–214. 10.1016/j.bbr.2015.08.010 26296669

[B90] Organisation for Economic Co-operation and Development (2017). *Obesity Update 2017*. Available online at: https://www.oecd.org/els/health-systems/Obesity-Update-2017.pdf (accessed September 30, 2020)

[B91] Pape H.-C. K. (2014). *Armin; Silbernagl, Stefan. Physiologie*. Stuttgart: Georg Thieme Verlag KG, 558–563.

[B92] Pasquali R. Vicennati V. Gambineri A. Pagotto U. (2008). Sex-dependent role of glucocorticoids and androgens in the pathophysiology of human obesity. *Int. J. Obes. (Lond)* 32 1764–1779. 10.1038/ijo.2008.129 18838976

[B93] Petry N. M. Barry D. Pietrzak R. H. Wagner J. A. (2008). Overweight and obesity are associated with psychiatric disorders: results from the national epidemiologic survey on alcohol and related conditions. *Psychosom. Med.* 70 288–297. 10.1097/PSY.0b013e3181651651 18378873

[B94] Preiss K. Brennan L. Clarke D. (2013). A systematic review of variables associated with the relationship between obesity and depression. *Obes. Rev. Official J. Int. Assoc. Study Obes.* 14 906–918. 10.1111/obr.12052 23809142

[B95] Puhl R. M. Andreyeva T. Brownell K. D. (2008). Perceptions of weight discrimination: prevalence and comparison to race and gender discrimination in America. *Int. J. Obes. (Lond)* 32 992–1000. 10.1038/ijo.2008.22 18317471

[B96] Puhl R. M. Brownell K. D. (2006). Confronting and coping with weight stigma: an investigation of overweight and obese adults. *Obesity* 14 1802–1815. 10.1038/oby.2006.208 17062811

[B97] Schroeder L. E. Leinninger G. M. (2018). Role of central neurotensin in regulating feeding: implications for the development and treatment of body weight disorders. *Biochim. Biophys. Acta Mol. Basis Dis.* 1864 900–916. 10.1016/j.bbadis.2017.12.036 29288794 PMC5803395

[B98] Schwartz M. B. Chambliss H. O. N. Brownell K. D. Blair S. N. Billington C. (2003). Weight bias among health professionals specializing in obesity. *Obes. Res.* 11 1033–1039. 10.1038/oby.2003.142 12972672

[B99] Service F. J. Jay J. M. Rizza R. A. O’Brien P. C. Go V. L. (1986). Neurotensin in diabetes and obesity. *Regul. Pept.* 14 85–92. 10.1016/0167-0115(86)90207-7 3715064

[B100] Spitzer R. L. Kroenke K. Williams J. B. (1999). Validation and utility of a self-report version of PRIME-MD: the PHQ primary care study. primary care evaluation of mental disorders. patient health questionnaire. *Jama* 282 1737–1744. 10.1001/jama.282.18.1737 10568646

[B101] Spitzer R. L. Kroenke K. Williams J. B. Lowe B. (2006). A brief measure for assessing generalized anxiety disorder: the GAD-7. *Arch. Int. Med.* 166 1092–1097. 10.1001/archinte.166.10.1092 16717171

[B102] Steele F. F. III Whitehouse S. C. Aday J. S. Prus A. J. (2017). Neurotensin NTS(1) and NTS(2) receptor agonists produce anxiolytic-like effects in the 22-kHz ultrasonic vocalization model in rats. *Brain Res.* 1658 31–35. 10.1016/j.brainres.2017.01.012 28089664

[B103] St-Gelais F. Jomphe C. Trudeau L.-E. (2006). The role of neurotensin in central nervous system pathophysiology: what is the evidence? *J. Psychiatry Neurosci. JPN* 31 229–245. 10.1016/j.bbadis.2017.12.036 16862241 PMC1488904

[B104] Stoschus B. Hamscher G. Ikonomou S. Partoulas G. Eberle C. Sauerbruch T. (1998). Effect of omeprazole treatment on plasma concentrations of the gastric peptides, xenin, gastrin and somatostatin, and of pepsinogen. *J. Pept. Res.* 52 27–33. 10.1111/j.1399-3011.1998.tb00649.x 9716248

[B105] Strack A. M. Sebastian R. J. Schwartz M. W. Dallman M. F. (1995). Glucocorticoids and insulin: reciprocal signals for energy balance. *Am. J. Physiol.* 268(1 Pt 2) R142–R149. 10.1152/ajpregu.1995.268.1.R142 7840315

[B106] Swanson S. A. Crow S. J. Le Grange D. Swendsen J. Merikangas K. R. (2011). Prevalence and correlates of eating disorders in adolescents. Results from the national comorbidity survey replication adolescent supplement. *Arch. Gen. Psychiatry* 68 714–723. 10.1001/archgenpsychiatry.2011.22 21383252 PMC5546800

[B107] Tanaka K. Masu M. Nakanishi S. (1990). Structure and functional expression of the cloned rat neurotensin receptor. *Neuron* 4 847–854. 10.1016/0896-6273(90)90137-5 1694443

[B108] Thiel A. Jacobi C. Horstmann S. Paul T. Nutzinger D. O. Schussler G. (1997). [A German version of the eating disorder inventory EDI-2]. *Psychother. Psychosom. Med. Psychol.* 47 365–376. 9411465

[B109] Tilg H. Moschen A. R. (2006). Adipocytokines: mediators linking adipose tissue, inflammation and immunity. *Nat. Rev. Immunol.* 6 772–783. 10.1038/nri1937 16998510

[B110] Torres S. J. Nowson C. A. (2007). Relationship between stress, eating behavior, and obesity. *Nutrition* 23 887–894. 10.1016/j.nut.2007.08.008 17869482

[B111] Tronieri J. S. Wurst C. M. Pearl R. L. Allison K. C. (2017). Sex differences in obesity and mental health. *Curr. Psychiatry Rep.* 19:29. 10.1007/s11920-017-0784-8 28439762

[B112] van de Sande-Lee S. Cardoso A. R. Garlipp C. R. Chaim E. A. Pareja J. C. Geloneze B. (2013). Cerebrospinal fluid xenin levels during body mass reduction: no evidence for obesity-associated defective transport across the blood-brain barrier. *Int. J. Obes.* 37 416–419. 10.1038/ijo.2012.70 22546776

[B113] Weiss H. Labeck B. Klocker J. Nehoda H. Mittermair R. Aigner F. (2001). Effects of adjustable gastric banding on altered gut neuropeptide levels in morbidly obese patients. *Obes. Surg.* 11 735–739. 10.1381/09608920160558687 11775572

[B114] Wice B. M. Reeds D. N. Tran H. D. Crimmins D. L. Patterson B. W. Dunai J. (2012). Xenin-25 amplifies GIP-mediated insulin secretion in humans with normal and impaired glucose tolerance but not type 2 diabetes. *Diabetes* 61 1793–1800. 10.2337/db11-1451 22522617 PMC3379667

[B115] Wice B. M. Wang S. Crimmins D. L. Diggs-Andrews K. A. Althage M. C. Ford E. L. (2010). Xenin-25 potentiates glucose-dependent insulinotropic polypeptide action via a novel cholinergic relay mechanism. *J. Biol. Chem.* 285 19842–19853. 10.1074/jbc.M110.129304 20421298 PMC2888395

[B116] Williams G. Cardoso H. Lee Y. C. Ghatei M. A. Flatt P. R. Bailey C. J. (1991b). Reduced hypothalamic neurotensin concentrations in the genetically obese diabetic (obob) mouse: possible relationship to obesity. *Metabolism* 40 1112–1116. 10.1016/0026-0495(91)90139-n 1943736

[B117] Williams G. Cardoso H. Lee Y. C. Ghatei M. A. Flatt P. R. Bailey C. J. (1991a). Reduced hypothalamic neurotensin concentrations in the genetically obese diabetic (ob/ob) mouse: possible relationship to obesity. *Metabolism* 40 1112–1116. 10.1016/0026-0495(91)90139-n 1943736

[B118] World Health Organization (2017). *Prevalence of obesity, age-standardized*. Available online at: https://www.who.int/data/gho/data/themes/topics/topic-details/GHO/prevalence-of-obesity-age-standardized (accessed September 30, 2020)

[B119] World Health Organization (2020). *Obesity and Overweight*. Available online at: https://www.who.int/news-room/fact-sheets/detail/obesity-and-overweight (accessed September 30, 2020)

[B120] Yawata Y. Yamatani K. Tominaga M. Ebitani I. Hara M. Sasaki H. (1984). Hyperglycemic effect of neurotensin. *Tohoku J. Exp. Med.* 143 185–196. 10.1620/tjem.143.185 6147906

[B121] Yip R. G. Wolfe M. M. (2000). GIP biology and fat metabolism. *Life Sci.* 66 91–103. 10.1016/s0024-3205(99)00314-8 10666005

