# Supplementary material for: Neurotensin and Xenin Show Positive Correlations With Perceived Stress, Anxiety, Depressiveness and Eating Disorder Symptoms in Female Obese Patients
Source: Front Behav Neurosci. 2021 Feb 16;15:629729. doi: 10.3389/fnbeh.2021.629729 (PMC7921165; doi:10.3389/fnbeh.2021.629729)
Supplement: Supplementary file 1 [file Data_Sheet_1.PDF]

Supplemental Table 1. Multivariable linear regression for neurotensin and xenin (dependent variables) in men and women

**Independent Variables**	**Men**		**Women**	
	**Neurotensin**	**Xenin**	**Neurotensin**	**Xenin**
Binge eating disorder<br>Sleep-associated breathing disorder<br>Arterial hypertension<br>Hypercholesterinemia<br>Hypertriglyceridemia<br>Fatty liver disease	R^2_{cor} = 0.071<br>p = 0.141	R^2_{cor} = 0.073<br>p = 0.135	R^2_{cor} = -0.013<br>p = 0.550	R^2_{cor} = -0.006<br>p = 0.484
Age (years)<br>PSQ-20 total score	R^2_{cor} = -0.002<br>p = 0.396	R^2_{cor} = -0.014<br>p = 0.572	R^2_{cor} = 0.033<br>p = 0.078	R^2_{cor} = 0.044<br>$\mathbf{p = 0.047}$
Age (years)<br>GAD-7 total score	R^2_{cor} = -0.020<br>p = 0.679	R^2_{cor} = -0.003<br>p = 0.412	R^2_{cor} = 0.500<br>$\mathbf{p = 0.034}$	R^2_{cor} = 0.046<br>$\mathbf{p = 0.043}$
Age (years)<br>PHQ-9 total score	R^2_{cor} = -0.018<br>p = 0.647	R^2_{cor} = -0.008<br>p = 0.476	R^2_{cor} = 0.060<br>$\mathbf{p = 0.021}$	R^2_{cor} = 0.037<br>p = 0.065
Age (years)<br>EDI-2 total score	R^2_{cor} = 0.015<br>p = 0.240	R^2_{cor} = -0.001<br>p = 0.386	R^2_{cor} = 0.059<br>$\mathbf{p = 0.022}$	R^2_{cor} = 0.049<br>$\mathbf{p = 0.038}$

Coefficients of determination (R^2_{cor}) were assessed using multivariable linear regression. Significant p-values are indicated in bold. Adjusted coefficient of determination is indicated as marker for the regression. Abbreviations: EDI-2, Eating Disorder Inventory-2; GAD-7, Generalized Anxiety Disorder-7; PHQ-9, Patient Health Questionnaire-9; PSQ-20, Perceived Stress Questionnaire-20.
