# Supplementary material for: Neurotensin and Xenin Show Positive Correlations With Perceived Stress, Anxiety, Depressiveness and Eating Disorder Symptoms in Female Obese Patients
Source: Front Behav Neurosci. 2021 Feb 16;15:629729. doi: 10.3389/fnbeh.2021.629729 (PMC7921165; doi:10.3389/fnbeh.2021.629729)
Supplement: Supplementary file 2 [file Data_Sheet_2.PDF]

**Supplemental Table 2.** Simple linear regression for neurotensin and xenin (dependent variables) in men and women

| Independent Variable | Men | | Women | |
|---|---|---|---|---|
| | **Neurotensin** | **Xenin** | **Neurotensin** | **Xenin** |
| Proton-pump inhibitor | $R^2_{cor}$ = -0.015<br>$p$ = 0.796 | $R^2_{cor}$ = -0.016<br>$p$ = 0.891 | $R^2_{cor}$ = -0.010<br>$p$ = 0.891 | $R^2_{cor}$ = 0.008<br>$p$ = 0.194 |
| Non-steroidal anti-inflammatory drugs | $R^2_{cor}$ = -0.016<br>$p$ = 0.983 | $R^2_{cor}$ = -0.012<br>$p$ = 0.625 | $R^2_{cor}$ = -0.011<br>$p$ = 0.940 | $R^2_{cor}$ = -0.008<br>$p$ = 0.616 |
| Fatty liver disease | $R^2_{cor}$ = -0.007<br>$p$ = 0.401 | $R^2_{cor}$ = -0.022<br>$p$ = 0.756 | $R^2_{cor}$ = -0.011<br>$p$ = 0.638 | $R^2_{cor}$ = 0.011<br>$p$ = 0.182 |

Coefficients of determination ($R^2_{cor}$) were assessed using simple linear regression. Significant $p$-values are indicated in bold. Adjusted coefficient of determination is indicated as marker for the regression.
